# Antidiabetic Effects of Gegen Qinlian Decoction via the Gut Microbiota Are Attributable to Its Key Ingredient Berberine

**DOI:** 10.1016/j.gpb.2019.09.007

**Published:** 2020-12-24

**Authors:** Xizhan Xu, Zezheng Gao, Fuquan Yang, Yingying Yang, Liang Chen, Lin Han, Na Zhao, Jiayue Xu, Xinmiao Wang, Yue Ma, Lian Shu, Xiaoxi Hu, Na Lyu, Yuanlong Pan, Baoli Zhu, Linhua Zhao, Xiaolin Tong, Jun Wang

**Affiliations:** 1 CAS Key Laboratory of Pathogenic Microbiology and Immunology, Institute of Microbiology, Chinese Academy of Sciences, Beijing 100101, China; 2 University of Chinese Academy of Sciences, Beijing 100049, China; 3 Department of Endocrinology, Guang'anmen Hospital, China Academy of Chinese Medical Sciences, Beijing 100053, China; 4 Beijing University of Chinese Medicine, Beijing 100029, China; 5 Laboratory of Protein and Peptide Pharmaceuticals & Laboratory of Proteomics, Institute of Biophysics, Chinese Academy of Sciences, Beijing 100101, China; 6 Shenzhen Hospital, Guangzhou University of Chinese Medicine, Shenzhen 518034, China

**Keywords:** Gut microbiota, Type 2 diabetes mellitus, Traditional Chinese medicine, Berberine, Gegen Qinlian Decoction

## Abstract

**Gegen Qinlian Decoction** (GQD), a **traditional Chinese medicine** (TCM) formula, has long been used for the treatment of common metabolic diseases, including **type 2 diabetes mellitus**. However, the main limitation of its wider application is ingredient complexity of this formula. Thus, it is critically important to identify the major active ingredients of GQD and to illustrate mechanisms underlying its action. Here, we compared the effects of GQD and **berberine**, a hypothetical key active pharmaceutical ingredient of GQD, on a diabetic rat model by comprehensive analyses of **gut microbiota**, short-chain fatty acids, proinflammatory cytokines, and ileum transcriptomics. Our results show that berberine and GQD had similar effects on lowering blood glucose levels, modulating gut microbiota, inducing ileal gene expression, as well as relieving systemic and local inflammation. As expected, both berberine and GQD treatment significantly altered the overall gut microbiota structure and enriched many butyrate-producing bacteria, including *Faecalibacterium* and *Roseburia*, thereby attenuating intestinal inflammation and lowering glucose. Levels of short-chain fatty acids in rat feces were also significantly elevated after treatment with berberine or GQD. Moreover, concentration of serum proinflammatory cytokines and expression of immune-related genes, including *Nfkb1*, *Stat1*, and *Ifnrg1*, in pancreatic islets were significantly reduced after treatment. Our study demonstrates that the main effects of GQD can be attributed to berberine via modulating gut microbiota. The strategy employed would facilitate further standardization and widespread application of TCM in many diseases.

## Introduction

Type 2 diabetes mellitus (T2DM) is a common metabolic disease characterized by low-grade inflammation, insulin resistance, and pancreatic islet beta cell dysfunction [1]. Currently, it is estimated that there are 425 million people with diabetes worldwide. With an ever-increasing rate of occurrence [2], T2DM poses a growing burden on the economy and medical expenditure globally. The treatments for T2DM are ancient and make up a significant proportion of traditional Chinese medicine (TCM) [3], [4], [5], [6], [7], [8], [9]. A large number of evidence-based clinical studies have shown the efficacy of several traditional or modified formulas composed of herbal substances [3], [4]. In addition to being cheaper than conventional treatment schemes, these formulas also exhibit higher efficacy or cause fewer adverse effects.

In recent years, new lines of evidence have also implied a vital role of gut microbiota in the pathophysiology of diabetes [10], [11], [12]. Shifts in the gut microbial composition and functions in T2DM patients in comparison with healthy individuals have been reported in multiple studies, for instance, decrease in *Bifidobacterium*, *Faecalibacterium*, and other microbial taxa associated with the production of butyrate [13], [14]. Accordingly, treatments of diabetes either with diet modifications or with medications are shown to be accompanied by changes in gut microbiota [15], [16], [17]. Accumulating evidence suggests that the microbiota changes could underlie the attenuation of T2DM. Metformin, the most widely used oral medication against T2DM, modulates the composition of gut microbiota and increases butyrate producers such as *Akkermansia* and *Blautia* [18], [19], which closely relates to its blood glucose-lowering effect in T2DM patients.

Gegen Qinlian Decoction (GQD) is a TCM formula, which comprises seven herbs, including *Rhizoma coptidis*, *Radix scutellariae*, *Radix puerariae*, *Rhizoma anemarrhenae*, *Radix panacis uinquefolia*, *Radix paeoniae rubra*, and *Rhizoma zingiberis*. We have previously demonstrated that GQD can be used effectively in the prevention and treatment of diabetes [3], [4], [5], [6], [9], potentially by inducing changes in intestinal microbiota and promoting beneficial taxa, including *Faecalibacterium*, *Bifidobacterium*, and *Gemmiger* [4], [9]. However, the complexity of most TCM mixtures in terms of chemical composition could hinder both their standardization and wider applications. The active pharmaceutical ingredients (APIs) of TCM can vary due to batch differences in herb quality, and understanding the mechanisms has also proven difficult for mixtures. Identifying the key API(s) could tremendously improve our understanding of medication and facilitate its application [20]. For example, metformin was originally derived from the traditional European herbal medicine *Galega officinalis* and was modified to reduce its side effects for wide use [21].

Following the same line of deduction and based on recent studies [19], [22], [23], [24], [25], we tested one particular potential API, berberine (BBR) [22], [26], and compared the efficacy of GQD *vs.* BBR alone. We hypothesized that the effect of GQD on T2DM could be recapitulated by BBR and exerted by modulating gut microbiota. We chose Goto-Kakizaki (GK) rats as the model for spontaneous, nonobese T2DM [27]. By investigating the efficacy of the two treatments, the structure of the gut microbiota, and the transcriptome of ileum tissue, we showed that the efficacy of GQD might be attributed primarily to its key ingredient, BBR, which is likely to alleviate T2DM via modulation of the gut microbiota, thereby reducing systemic and local inflammation. Our study would facilitate the standardization and application of GQD and potentially other TCM formulas and also add to the current body of knowledge about BBR, a promising new drug for the treatment of T2DM.

## Results

### GQD and BBR treatments can alleviate diabetes in a rat model

**Figure 1:**
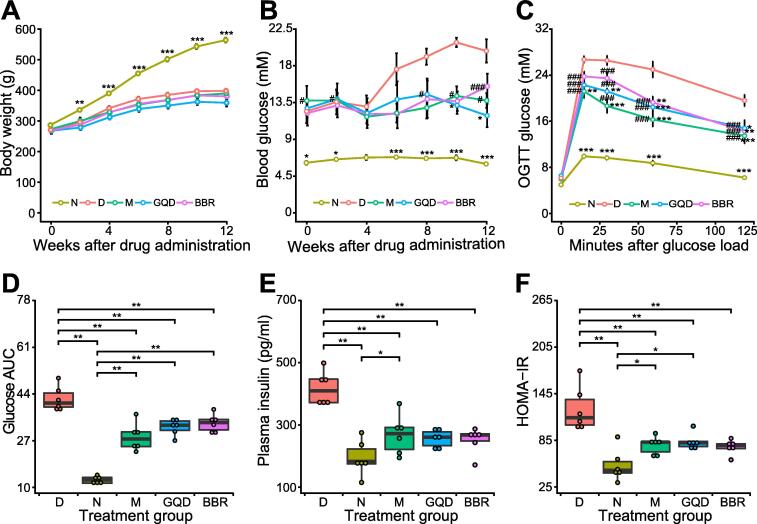
**Effects of different drug treatments on rat metabolism in each group** **A.** Time course of changes in body weight. **B.** Time course of changes in nonfasting blood glucose level. Rat body weight and nonfasting blood glucose level were measured every 2 weeks during the 12-week experiment. **C.** Time course of changes in blood glucose profile. After 12 h of fasting, OGTT was conducted in the rats 1 week before euthanasia, and blood glucose level was measured at 0, 15, 30, 60, and 120 min after oral administration of glucose at a dose of 2 g/kg. **D.** AUC of blood glucose during OGTT in different treatment groups. **E.** Fasting plasma insulin level measured at time = 0 min. **F.** HOMA-IR measured at time = 0 min. Data are presented as mean ± SEM (*n* = 6 samples per group). Two-way ANOVA followed by Tukey’s HSD post hoc test was performed to assess differences in body weight and blood glucose (*, *P* < 0.05 *vs.* D group; ^#^, *P* < 0.05 *vs.* N group). The Kruskal–Wallis test followed by the pairwise Wilcoxon rank sum test was used to examine the significance of differences in AUC, insulin level, and HOMA-IR (*, *P* < 0.05; **, *P* < 0.01; ***, *P* < 0.001). N, normal; D, diabetes; M, metformin; GQD, Gegen Qinlian Decoction; BBR, berberine; OGTT, oral glucose tolerance test; AUC, area under the curve; HOMA-IR, homeostatic model assessment for insulin resistance.

To explore the effects of different treatments on rats, we monitored and analyzed the changes in blood glucose level and body weight for 12 weeks. Compared to the normal Wistar rats (N group), the weight of GK rats was significantly lower at each time point during the experimental period [*P* < 0.05, Tukey’s honestly significant difference (HSD) test]. No significant differences were observed between the diabetes model group (D group) and the three drug-intervention groups, including the metformin (M), GQD, and BBR groups (Figure 1A). All GK rats had significantly higher nonfasting blood glucose (NFBG) than the N group during the experimental period (*P* < 0.05, Tukey’s HSD test). Although blood glucose level showed a slightly downward trend compared with the D group, no significant difference was observed in any drug treatment group. After 12 weeks, although all interventions achieved glucose-lowering effects, the effect of BBR was slightly weaker than that of GQD (Figure 1B).

To determine the consequences of different treatments on the glucose metabolic status of rats, an oral glucose tolerance test (OGTT) was conducted one week before euthanasia. We found that glucose clearance capacity was improved markedly in the drug-treated groups, as reflected by the significantly lower peak glucose level (Figure 1C, *P* < 0.001, Wilcoxon rank sum test) and smaller area under the curve (AUC) values (Figure 1D, *P* < 0.01, Wilcoxon rank sum test), compared to the D group. Similarly, all drug-intervention groups also showed significantly reduced fasting plasma insulin level (Figure 1E, *P* < 0.01, Wilcoxon rank sum test) as well as homeostatic model assessment-insulin resistance (HOMA-IR) (Figure 1F, *P* < 0.01, Wilcoxon rank sum test).

### Community structure of gut microbiota shows distinct changes after different treatments

**Figure 2:**
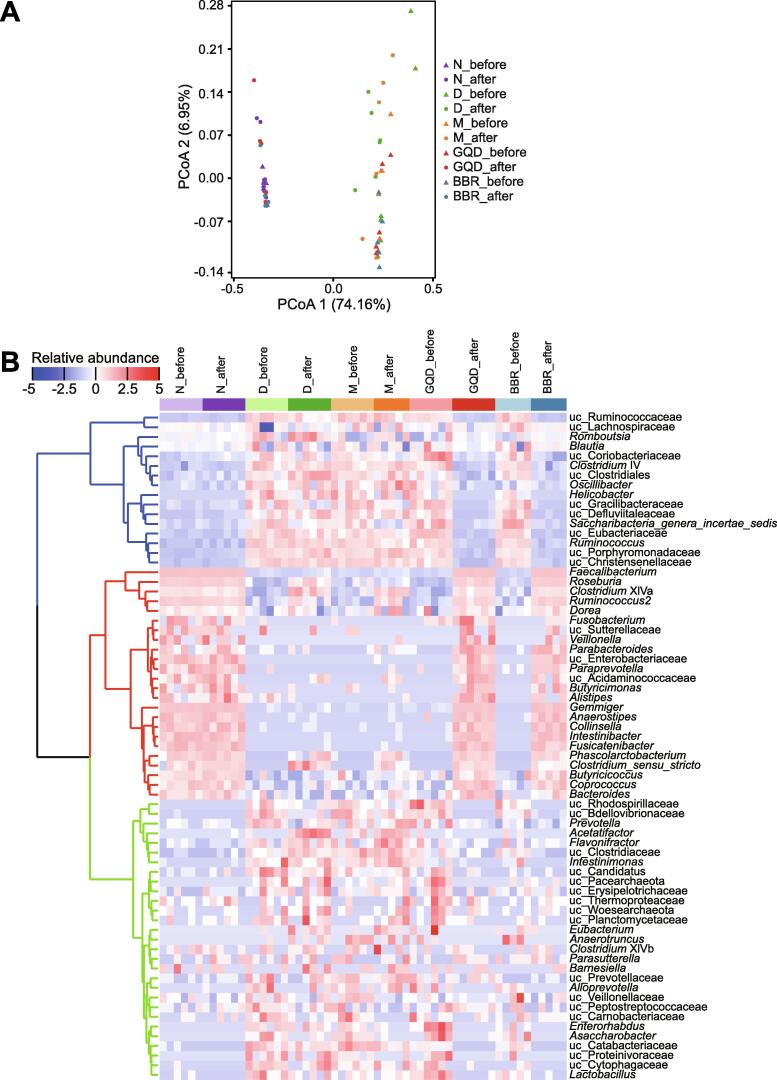
**Comparison of the overall microbial structure of gut microbiota in rats between different treatment groups** **A.** Bray–Curtis distance PCoA of the rat fecal microbiota before (denoted as triangles) and after different treatments (denoted as circles). A pairwise PERMANOVA test implemented in the *adonis* function in the *vegan* R package was used to test the effects of different treatments on the gut microbiota. **B.** Heatmap representing the hierarchical clustering based on the microbial composition at the genus level. Filtered genera with a prevalence of at least 30% in all samples were used for clustering. Samples from different groups are color-coded on the top of the panel. All genera were divided into three clusters, and each cluster was color-coded (blue: cluster 1, representing genera reduced in GQD and BBR groups; red: cluster 2, representing genera enriched in GQD and BBR groups; green: cluster 3, mainly composed of genera reduced in GQD and BBR groups). PCoA, principal coordinate analysis; uc, unclassified higher taxonomic level.

The gut microbiota structure before and after individual treatment showed significant shifts in the GQD (adjusted *P* = 0.008, PERMANOVA test with *adonis*) and BBR (adjusted *P* = 0.009) treatment groups, while no significant changes in the overall structure of the gut microbiota were observed in the N (adjusted *P* = 0.262), D (adjusted *P* = 0.073), or M group (adjusted *P* = 0.065). Specifically, in Bray–Curtis distance-based principal coordinate analysis (PCoA), the gut microbiota structure of the GQD and BBR groups showed a distinct deviation along PCoA1 (explaining 74.16% of the variation) after treatment, indicating significant changes in the core microbiota after treatments (Figure 2A). Interestingly, both the GQD and BBR treatment groups displayed extremely similar community structures that resembled the microbial structure of normal Wistar rats after treatment (GQD *vs.* BBR: adjusted *P* = 0.269; GQD *vs.* N: adjusted *P* = 0.855; BBR *vs.* N: adjusted *P* = 0.226). These results demonstrate that both GQD and BBR treatments can restore the gut microbiota of diabetic GK rats to that of normal Wistar rats, and BBR alone functions as the principal driver altering the rat gut microbiota.

Since the microbiota of GK rats under different drug treatments exhibited significant differences from the untreated D group, we next explored the variations before and after treatment both at the phylum and genus levels. At the phylum level, the gut microbiota before and after treatment was both largely dominated by two major phyla, Firmicutes and Bacteroidetes. Meanwhile, the Firmicutes/Bacteroidetes ratio also increased significantly after various treatments (Figure S1B, *P* < 0.01, Wilcoxon rank sum test). At the genus level, groups treated with GQD and BBR showed high similarity, and were indistinguishable from normal Wistar rats (Figure S1A). After treatment, both GQD and BBR groups were significantly enriched in *Faecalibacterium*, *Roseburia*, *Clostridium* XIVa, *Ruminococcus2*, and *Dorea*, as well as a number of much rarer genera, including *Parabacteroides*, *Paraprevotella*, *Butyricimonas*, *Alistipes*, *Gemmiger*, *Butyricicoccus*, and *Coprococcus*, according to clustering analysis of all genera based on changes in abundance (Figure 2B). In contrast, metformin treatment enriched a few genera partially overlapping with those in the GQD or BBR group, such as *Clostridium* XIVa, *Ruminococcus2*, *Dorea*, *Clostridium sensu stricto*, and *Phascolarctobacterium*. However, we also observed that the D group had a similar genus enrichment profile as the M group, indicating that these genera are highly likely to be enriched due to the maturity of gut flora in GK rats over time, regardless of metformin treatment. Moreover, BBR treatment significantly decreased the alpha diversity of the gut microbiota (*P* < 0.01, Wilcoxon rank sum test), while GQD only decreased the Shannon evenness index (Figure S2). These data show that both GQD and BBR treatments markedly alter the gut microbiota structure and enrich a number of genera, predominantly butyrate-producing bacteria, including *Faecalibacterium*, *Roseburia*, and *Clostridium* XIVa. More importantly, BBR, as one of the APIs of GQD, achieved almost the same effect as GQD in altering the gut microbiota structure of rats.

**Figure 3:**
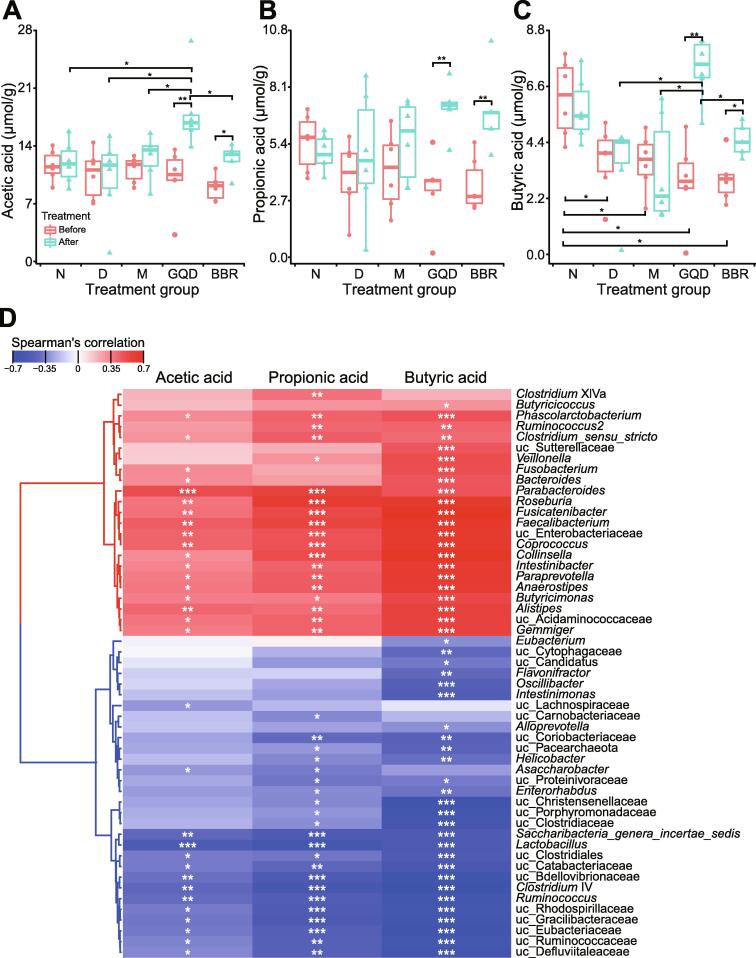
**Changes in SCFA levels in the feces of rats after different treatments** **A.** Concentration of acetic acid in feces of rats from different groups before and after treatment. **B.** Concentration of propionic acid in feces of rats from different groups before and after treatment. **C.** Concentration of butyric acetic in feces of rats from different groups before and after treatment. **D**. Spearman’s correlation coefficient of microbial genera abundance and SCFAs was visualized in a hierarchically clustered heatmap. Red indicates a positive correlation and blue a negative correlation. The Kruskal–Wallis test followed by the pairwise Wilcoxon rank sum test was applied to compare different untreated or treated groups. The Wilcoxon rank sum test was used to compare a group before and after treatment. Differences with *P* < 0.05 (or BH adjusted *P* < 0.05) were considered to be significant. *, *P* < 0.05; **, *P* < 0.01. *n* = 5–6 samples per group. SCFA, short-chain fatty acid; BH, Benjamini–Hochberg.

To confirm whether the enriched genera really increased the levels of butyrate, we next measured the fecal concentration of short-chain fatty acids (SCFAs). After treatment, a significant increase in acetic acid, propionic acid, and butyric acid was observed in both the GQD and BBR treatment groups (Figure 3, *P* < 0.01, Wilcoxon rank sum test). The GQD group had significantly higher levels of acetic acid and butyric acid than any other group (*P* < 0.05, Wilcoxon rank sum test). We also found no significant differences in the levels of acetic acid or propionic acid among all groups (Figure 3A and B), while the N group had significantly higher butyric acid levels than all GK rat groups before treatment (Figure 3C, *P* < 0.05, Wilcoxon rank sum test). Moreover, we examined the correlation between the levels of SCFAs and the relative abundance of microbial genera. Concentration of SCFAs, especially butyric acid, was positively correlated with several aforementioned enriched genera and negatively correlated with some depleted bacteria that are potentially pathogenic, such as *Helicobacter*. Profiles of these SCFAs were highly consistent with the changes in gut microbial composition of the corresponding groups, indicating that genera enriched by GQD or BBR treatments probably increase the production of SCFAs.

### Gene expression profiles of rat ileum are related to treatment schemes

Previous studies have shown that gut microbiota could modulate gene expression in the gastrointestinal epithelium [28], [29], [30], [31], [32]. Given that gut microbiota of rats treated with GQD or BBR was highly similar in both the community structure and composition, we next aimed to determine through transcriptomic analysis whether the gene expression profiles of rat ileum were also similar between different treatment groups. As expected, principal component analysis showed that the N group had distinct gene expression signatures compared with the other GK rat groups and were separated from the other groups along PC1 (explaining 26.94% of variation) (Figure S3A). Furthermore, the GQD and BBR treatment groups displayed relatively similar gene expression profiles. These results are consistent with the corresponding gut microbiota structure as well as the outcomes of treatment for T2DM, supporting the essential roles of gut microbiota in modulating transcription in the rat ileum.

Next, we set out to identify genes that were differentially expressed through pairwise comparisons of groups (*P* < 0.05). Compared with the N group, the number of differentially expressed genes (DEGs) in the D, M, GQD, and BBR groups was 518 (189/329; upregulated and downregulated DEGs, respectively), 441 (181/260), 1156 (545/611), and 1063 (394/669), respectively (Figure S3B–E, Table S2). Pairwise comparisons for differential expression within the four GK rat groups contributed to an additional 855 unique DEGs. Interestingly, the GQD and BBR groups displayed similar trends in the comparative analysis by volcano plot, and only 102 DEGs (the lowest number among all comparisons) were identified between these two groups, which implied a highly similar gene expression pattern between them (Figure S3F–K).

Next, we interpreted the sets of DEGs (in comparison with the N group) through Gene Ontology (GO) and Kyoto Encyclopedia of Genes and Genomes (KEGG) pathway enrichment analyses. We found that the DEGs between GK rats and Wistar rats were mainly involved in host immunity in both GO and KEGG analyses. However, there were many overlapping KEGG pathway entries related to immunity among the upregulated or downregulated DEGs, such as cell adhesion molecules (CAMs) as well as antigen processing and presentation pathways, reflecting the abnormal state of immune function in GK rats (Figure S4). Interestingly, the M group also showed a similar enrichment profile to the D group, indicating similar gene expression patterns between the M and D groups. Similarly, the terms enriched among the DEGs of the GQD and BBR groups were also highly similar, including immune response-related entries enriched among the downregulated genes and lipid metabolism-related entries enriched among the upregulated genes. Given the significant differences in gene expression between GK rats and Wistar rats, subsequent functional enrichment analysis was performed using the DEGs identified among the four groups using GK rats.

**Figure 4:**
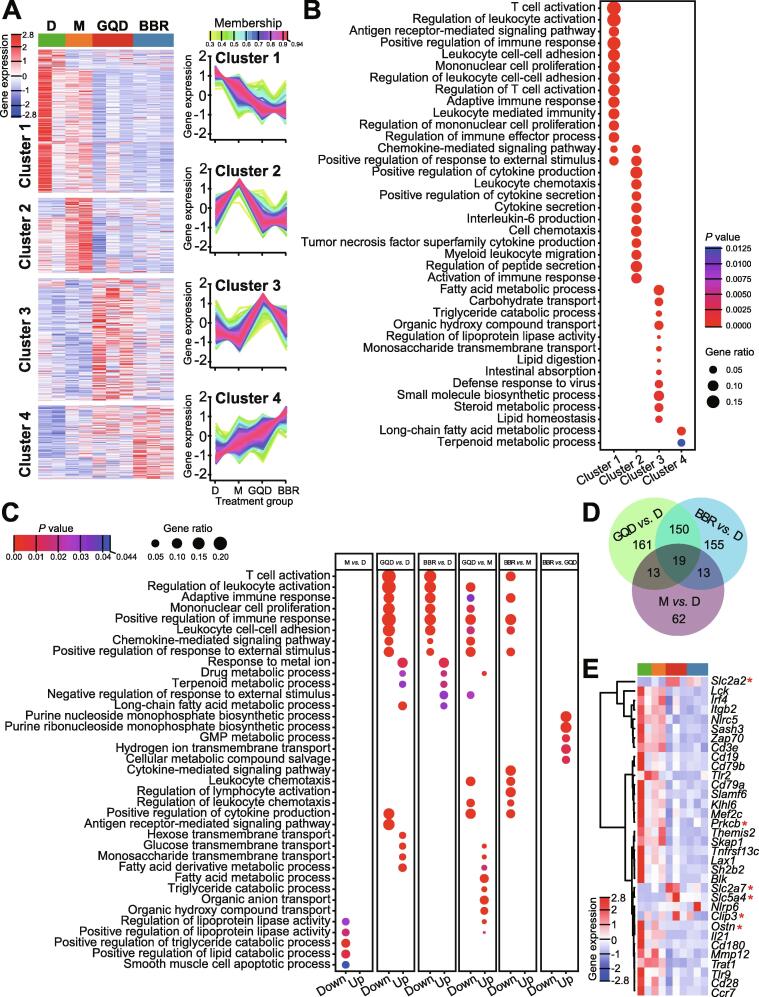
**Comparisons of gene expression profiles by RNA-seq among different treatment groups in the rat ileum** **A.** Genewise clustering heatmap of all 855 DEGs according to the gene expression patterns, showing segregation into four clusters. Cluster 1 (*n* = 295) includes genes with reduced expression in GQD and BBR treatment groups. Cluster 2 (*n* = 156) includes genes with expression upregulated in M group and downregulated in GQD and BBR groups. Cluster 3 (*n* = 252) includes genes with expression upregulated in both GQD and BBR groups, but higher in GQD group. Cluster 4 (*n* = 152) includes genes with increased expression among all three treatment groups, with the highest expression levels in BBR group. **B.** Doptplot of GO terms enriched for the four clusters. **C.** GO terms enriched among the upregulated DEGs and downregulated DEGs between the indicated groups (M *vs.* D; GQD *vs.* D; BBR *vs.* D; GQD *vs.* M; BBR *vs.* M; BBR *vs.* GQD). All *P* values in the GO enrichment analysis were adjusted for multiple testing using the BH method. Adjusted *P* < 0.05 was considered significant. Dot size represents the ratio of the number of DEGs to the number of genes in the corresponding entry. Dots are color-coded according to the adjusted *P* value for each term. **D.** Venn diagram displaying the shared DEGs between the three treatment groups. **E.** Heatmap of the representative DEGs that are shared by GQD *vs.* D and BBR *vs.* D comparisons and enriched for the GO terms “positive regulation of immune response” (GO:0050778) or “carbohydrate transport” (GO:0008643, denoted with a red asterisk), including immune and metabolic genes. DEG, differentially expressed gene; GO, Gene Ontology.

Based on the genewise expression profiles across all samples, we then grouped the 855 DEGs identified between the four GK rat groups into four clusters using the *TCseq* tool (Figure 4A, Table S3). These four clusters were functionally annotated by GO enrichment analysis (Figure 4B) and KEGG pathway enrichment analysis (Figure S5A, Table S3). We found that cluster 1 gene sets were mainly enriched in immune regulation-related terms, such as T cell activation (*Cd3d*, *Cd3e*, *Cd8a*, *Fyn*, *Lck*, *Zap70*, *Cd28*, *etc*.), whereas cluster 2 was enriched in terms associated with cytokine production and secretion (*Ccr5*, *Irf4*, *Il17a*, *Tlr8*, *Foxp3*, *Il17f*, *etc*.). Interestingly, we found that gene sets in clusters 1 and 2 mainly comprised genes with significantly downregulated expression in both the GQD and BBR groups, implying a potential role for GQD or BBR treatments in alleviating inflammation. Enriched terms for cluster 3 genes are related to lipid and carbohydrate metabolism, such as fatty acid metabolic process (*Apoa1*, *Apoa4*, *Apoa5*, *Apoc3*, *Angptl4*, *Cyp2b1*, *Cyp2d2*, *Cyp2d3*, *Cyp2d5*, *Cyp4a1*, *Slc27a2*, *Adh7*, *etc*.) and carbohydrate transport (*Slc2a2*, *Slc2a5*, *Slc2a7*, *Slc5a1*, *Slc5a4*, *Slc5a9*, *Aqp1*, *Aqp7*, *Lct*, *etc*.). Cluster 4 genes are also enriched in lipid metabolism-related terms, such as long-chain fatty acid metabolic process (*Cyp2b2*, *Cyp2c23*, *Cyp2c6v1*, *Cyp2j4*, *Cyp4f18*, *Cd36*, *Pnpla3*, *etc*.). Given that cluster 3 gene sets showed relatively high expression in the GQD group, whereas cluster 4 gene sets showed higher expression in the BBR group, these results suggest that lipid and carbohydrate metabolism could be improved after GQD or BBR treatments. Interestingly, earlier studies have shown that expression of *Angptl4*, the gene encoding angiopoietin like 4 that strongly inhibits the activity of lipoprotein lipase and promotes the cellular uptake of triglycerides, is regulated by gut microbiota [30], [32], [33], [34], [35]. The upregulated expression of *Angptl4* was also consistent with the slight weight loss effect of both GQD and BBR treatments in comparison with the D group, although non-significantly (Figure 1A).

Moreover, we conducted enrichment analysis using upregulated and downregulated DEGs of each group (identified by pairwise comparisons among the four GK rat groups). As shown in Figure 4C and Figure S5B, both GQD and BBR treatments significantly downregulated the immune response and upregulated the response to metal ions (*P* < 0.05, Fisher’s exact test). These findings are consistent with the enrichment analysis by the genewise clustering method shown above. In addition, through comparative transcriptomic analysis between these four groups, we observed that very few GO terms were enriched in the M *vs.* D, and BBR *vs.* GQD comparisons, suggesting that the effects of metformin treatment on the host are not as evident as those of GQD or BBR treatments, while GQD and BBR treatments exert highly similar effects on the GK rats. We also analyzed the 102 upregulated and downregulated DEGs between the GQD and BBR treatment groups and found five significantly enriched GO terms (*P* < 0.05, Fisher’s exact test). These include “purine nucleoside monophosphate biosynthetic process”, “purine ribonucleoside monophosphate biosynthetic process”, “GMP metabolic process”, “hydrogen ion transmembrane transport”, and “cellular metabolic compound salvage”. Interestingly, these five terms are exclusively represented by upregulated genes (Figure 4C). No KEGG pathways were enriched (Figure S5B).

Additionally, we were able to identify 169 common DEGs between the GQD and BBR groups (each in comparision to the D group). Almost half of the DEGs in both comparisons were shared (GQD: 49.3%; BBR: 50.1%, Figure 4D). Interestingly, all 169 common DEGs except *Unc93a* showed extremely concordant gene expression patterns (downregulated or upregulated compared with the D group, Table S2), suggesting that GQD and BBR have highly similar treatment effects. GO and KEGG pathway enrichment analyses of these 169 DEGs also identified some entries related to inflammation (Figure S6, Table S2). We further examined the shared genes from the significantly enriched GO terms (GO:0050778 “positive regulation of immune response” and GO:0008643 “carbohydrate transport”) (Figure 4E). We found that expression of genes enriched in the “positive regulation of immune response” was significantly downregulated, whereas expression of genes enriched in “carbohydrate transport” was upregulated, implying that GQD and BBR might reduce inflammation through similar mechanisms.

Together, our RNA-seq data reveal that traditional Chinese herbal medicine treatment significantly alleviates the inflammation of the GK rat ileum and improves the metabolism of lipids and carbohydrates, potentially through the alteration in the gut microbiota.

### GQD and BBR treatments attenuate systemic and local pancreatic islet inflammation

**Figure 5:**
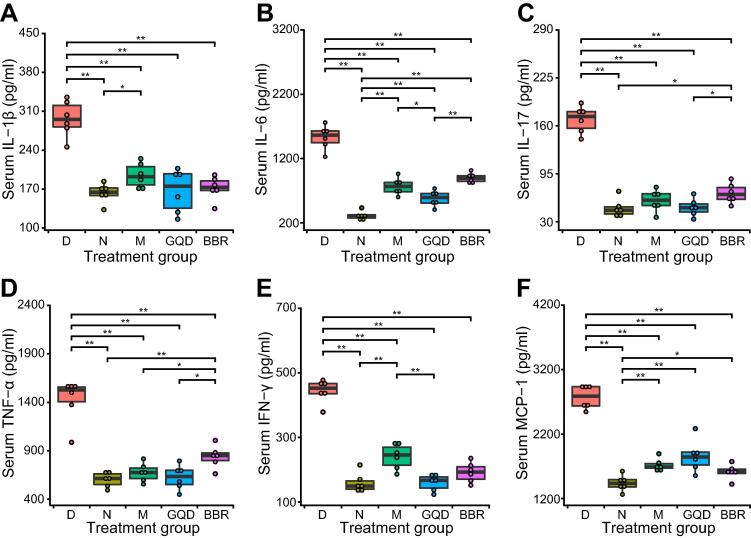
**Effects of different treatments on proinflammatory cytokine concentrations in the serum** Concentrations of six representative cytokines, IL-1β (**A**), IL-6 (**B**), IL-17 (**C**), TNF-α (**D**), IFN-γ (**E**), and MCP-1 (**F**), in the serum of rats were assessed after various treatments. The Kruskal–Wallis test followed by the pairwise Wilcoxon rank sum test was applied to test the differences in cytokine levels among different groups. *, *P* < 0.05; **, *P* < 0.01. *n* = 6 samples per group.

To explore whether inflammation in GK rats is alleviated after drug treatment, we further evaluated the inflammation status of the rats by measuring the levels of several indicator proinflammatory cytokines. We found that the levels of all six cytokines detected, including interleukin 1β (IL-1β), IL-6, IL-17, tumor necrosis factor α (TNF-α), interferon γ (IFN-γ), and monocyte chemoattractant protein 1 (MCP-1), were significantly reduced in all drug-intervention groups (*P* < 0.01, Wilcoxon rank sum test), implying systematically ameliorated inflammation (Figure 5). Interestingly, among the three treatment groups, the lowest levels of these proinflammatory cytokines except MCP-1 were found in rats after GQD treatment.

**Figure 6:**
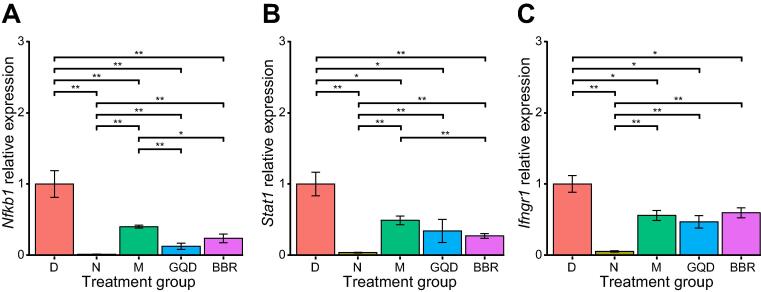
**Analysis of relative expression of immune-related genes in isolated rat islet beta cells using qPCR** Expression levels of three candidate genes, *Nfkb1* (**A**), *Stat1* (**B**), and *Ifngr1* (**C**), were measured. All data are presented as mean ± SEM; *n* = 6 samples per group. The Kruskal–Wallis test followed by the pairwise Wilcoxon rank sum test was applied to test the differences in gene expression levels among the five groups. *, *P* < 0.05; **, *P* < 0.01. *n* = 6 samples per group.

In addition, we investigated the effects of these treatments on local pancreatic islet inflammation by checking the expression levels of key immune-related genes (*Nfkb1*, *Stat1*, and *Ifngr1*) through qPCR. Compared to the N group, the expression levels of *Nfkb1*, *Stat1*, and *Ifngr1* were significantly elevated in the D group (*P* < 0.01, Wilcoxon rank sum test). After drug treatment, expression of these genes was significantly downregulated in all groups (*P* < 0.01, Wilcoxon rank sum test). Moreover, the GQD and BBR groups showed relatively lower expression levels of *Nfkb1* and *Stat1* but similar levels of *Ifngr1* compared with the M group (Figure 6). Given the important roles of these three genes in the inflammatory response, these results show that treatment with metformin, GQD, or BBR can alleviate the local inflammation of islet cells, thereby improving diabetes status. Altogether, our results indicate that drug intervention could alleviate both systemic and local (pancreatic islet) inflammation.

## Discussion

Our previous studies suggest that the gut microbiota might serve as a potential drug target of GQD for the treatment of T2DM [4], [9]. However, it remains unclear which ingredient(s) of GQD could modulate the structure of the gut microbiota and how the altered flora could affect host metabolism. In the current study, we employed a deductive approach to identify the key API of GQD that could regulate gut microbiota. In view of the efficacy of BBR on diabetes and its role in modulating gut microbiota, we hypothesized that BBR, the known API of *Rhizoma coptidis*, could act as the major component of GQD to modulate gut microbiota. We then tested our hypotheses in GK rats, a nonobese diabetic animal model for spontaneous T2DM. Our results showed that both GQD and BBR effectively alleviated the symptoms of T2DM, enriched butyrate-producing bacteria, increased butyrate production, and reduced systemic and local (intestinal and pancreatic islet) inflammation. More importantly, we found that GQD and BBR showed similar efficacy in modulating gut microbiota and inducing ileal gene expression profiles, implying that BBR is the key API of GQD.

After 12 weeks of treatment, we found that all drug treatments ameliorated hyperglycemia and improved the capacity of glucose clearance and the HOMA-IR. GQD-treated rats showed a slightly better, although not significant, capacity for glucose clearance than their BBR-treated counterpart (Figure 1C and D). Interestingly, the GQD and BBR groups showed slightly lower body weight than the D group, although the differences were not significant (Figure 1A). This is consistent with a previous study showing that GQD and BBR could effectively prevent the development of obesity in high-fat diet-fed rats, which could be explained by the upregulated expression of *Angptl4* (also called *Fiaf*) in the ileum. Angiopoietin-like 4 can inhibit the activity of lipoprotein lipase and lead to decreased triglyceride deposition in adipocytes, thereby reducing the weight of rats [9], [19], [23], [25].

Recent studies have shown that gut microbiota plays an increasingly important role in the development and treatment of T2DM [4], [11], [15], [17], [36], [37], [38]. Both the first-line drug metformin and TCM can significantly alter the structure of the gut microbiota, thereby contributing to reduced blood glucose levels [4], [17], [38], [39], [40], [41]. In this study, no significant alterations of gut microbiota in normal Wistar rats or diabetic GK rats were observed before and after treatment, while there were distinct differences in microbial composition between these two rat groups, suggesting that the dysbiotic gut microbiota in diabetic rats is due to genetic differences. It is of note that metformin treatment resulted in minimal structural changes to the gut microbiota in GK rats, which is inconsistent with the role of metformin in shaping the gut microbiota in previous studies [17], [39], [40], [41]. This could be explained by the heterogeneity in the genetic background of the animal models used in different studies. For instance, Bauer et al. and Zhang et al. used Wistar and Sprague-Dawley rats in their studies to illustrate the regulatory effects of metformin on the gut microbiota, respectively [17], [19]. Bauer found that metformin shifts the upper small intestinal microbiota by increasing the abundance of *Lactobacillus*, thereby improving the glucoregulatory pathway of Wistar rats [17]. In the latter study, metformin exhibited an obviously lower capacity to modulate gut microbiota than BBR [19]. This also provides a reasonable explanation as to why metformin could not reverse the dysbiotic gut microbiota of GK rats. Moreover, we found that GQD and BBR treatments resulted in an extremely similar community structure and the gut microbiota of the treated diabetic rats was indistinguishable from that of normal rats. These data suggest that modulation of gut microbiota by GQD may be mainly due to BBR. Interestingly, we also observed that GQD and BBR treatments show minor differences in altering the alpha diversity of gut microbiota. The BBR group showed a significant decrease in all the alpha diversity metrices, whereas the GQD group only showed a significant decrease in the Shannon evenness index. This might reflect the complex interactions between multiple components within the GQD formula [42]. BBR has been shown to exhibit a broad spectrum of antibacterial activities, especially for opportunistic pathogens, including *Staphylococcus*, *Salmonella*, and *Pseudomonas* [23], while other unknown ingredients in GQD might exert opposite effects. Consistent with our findings, Lv et al. failed to observe significant alpha diversity changes of gut microbiota by GQD treatment in mice as well [43]. Once treated with GQD or BBR, we found that one particular group of bacteria was markedly enriched in the gut microbiota, including *Faecalibacterium*, *Roseburia*, *Clostridium* XIVa, *Ruminococcus2*, *Dorea*, *Butyricicoccus*, and *Coprococcus*. These bacteria are well-known butyrate producers that can act as probiotics to exert beneficial effects on the host [44], [45]. Consequently, increased production of fecal SCFAs, especially butyrate, was also detected in our study. Butyrate can reduce liver glucose production and improve insulin resistance via the gut-brain axis [46]. Additionally, it can serve as the preferred energy source for colon cells locally [44], enhance the gut barrier by promoting the expression of tight junction proteins [47] and mucins [48], and attenuate low-grade inflammation by activating anti-inflammatory Treg cells [49] and suppressing pathways involved in the production of proinflammatory cytokines and chemokines [50]. Thus, the increase in butyrate producers or butyric acid in the colon has been repeatedly demonstrated to be associated with lowering blood glucose and alleviating intestinal inflammation [50], [51]. However, we can only establish correlations between the gut microbiota and its metabolites in the treatment of diabetes in rat models, since the identification of causal relationships would require transplanting GQD- or BBR-treated microbiota into germ-free animals in future studies.

To determine the effects of significantly altered gut microbiota on host metabolism, we performed transcriptomic analysis of rat ileum from different treatment groups. Compared to normal rats, diabetic rats had distinct gene expression profiles, possibly due to differences in host genetics or aberrant microbial composition (Figure S3). Treatment with GQD or BBR significantly altered ileal gene expression by upregulating and downregulating specific sets of genes, while metformin treatment had a relatively small effect on the host transcriptome. Comparative transcriptomic analysis suggests that the expression profiles between the GQD and BBR groups are very similar, with a few DEGs identified. These two groups also shared many common DEGs, GO terms, and KEGG pathways. These results further confirm that BBR seems to act as the key API of GQD to exert its antidiabetic effects, and other components may play potentially synergistic but minor roles, such as enhancing carbohydrate transmembrane transport (Figure 4C). Among the GO terms and KEGG pathways that were significantly enriched by GQD or BBR treatment are those involved in inflammation, which were downregulated. Intestinal inflammation has been shown to be critical in the onset of T2DM [52], [53]. Reduction of the inflammatory response by GQD or BBR, whether directly by the drug itself, by gut microbiota shifts, or by the increased butyrate production, could contribute to the alleviation of T2DM. In addition, GQD or BBR treatment upregulated pathways involved in drug and retinol metabolism. Collectively, these data, in conjunction with previous results, suggest that BBR is the key API of GQD, exerting antidiabetic effects mainly by reducing intestinal inflammation.

To further confirm whether inflammation in rats was alleviated with drug treatment, we measured the serum levels of several proinflammatory cytokines at the protein level. We found that the levels of cytokines TNF-α, IFN-γ, MCP-1, IL-1β, IL-6, and IL-17 were all elevated in diabetic rats. All six cytokines were significantly reduced in all drug-intervention groups (M, GQD, and BBR), implying ameliorated systemic inflammation. These data confirm that changes in gut microbiota and reduced intestinal inflammation are closely related to the level of inflammation in the body. Although metformin does not alter the gut microbiota of GK rats, it can suppress the inflammatory response by inhibiting nuclear factor κB via AMP-activated protein kinase-dependent and -independent pathways [54]. Chronic low-grade inflammation could lead to insulin resistance in target organs, which is associated with glucose metabolism and the apoptosis and dysfunction of islet cells [14], [55]. We found that these treatments also reduced islet inflammation, marked by the downregulation of immune-related marker genes, such as *Nfkb1*, *Stat1*, and *Ifngr1*. Altogether, these data suggest that both GQD and BBR treatments can alleviate systemic and local (pancreatic islet) inflammation, potentially by modulating the gut microbiota and increasing the production of butyrate.

Our study has several limitations. First, we compared only the role of the hypothetical API (BBR) with GQD in alleviating diabetes. It cannot be ruled out that other abundant components in GQD, such as baicalin and puerarin, also exert synergistic antidiabetic effects. More comprehensive studies are needed to clarify the exact role of each component in this formula. In addition, despite the extremely low oral bioavailability of GQD and BBR [4], [19], the possibility that they directly affect host tissues rather than modulating the gut microbiota also exists. By analyzing germ-free animals treated with GQD or BBR, we should be able to examine the mechanisms by which drugs or gut microbiota influence host physiology.

In conclusion, we have confirmed that BBR may play the primary role of the TCM formula, GQD, for the treatment of T2DM. This does not exclude the possibility that other components of GQD possess the same properties. A more complex and time-consuming screening process would be needed to fully understand the complete spectrum of components included in this complex formula. As a compound with properties in modulating gut microbiota, BBR has great potential for treating diabetes, obesity, as well as other diseases, and can serve as a candidate drug for a wider range of clinical applications in the future. Our analyses also indicate that GQD and BBR potentially alleviate systemic and local inflammation by enriching butyrate-producing bacteria, thereby ameliorating diabetes. Finally, our study provides a feasible approach to standardizing TCM formulas by identifying and functionally investigating potentially key APIs, reducing its complexity to avoid the unintended side effects of unknown components, and illustrating its main mechanisms of action.

## Materials and methods

### Reagents

GQD was purchased from Sichuan New Green Pharmaceutical Technology Company (Catalog No. QHCDX3c4; Chengdu, China). BBR (Catalog No. B21379) and metformin (Catalog No. B25331) were both purchased from Yuanye Biotechnology Company (Shanghai, China).

### Animals

Male GK rats (Catalog No. NBDC00680; RIKEN BioResource Center, Ibaraki, Japan) and Wistar rats (Catalog No. 102; Vital River Laboratory Animal, Beijing, China), all at 6 weeks of age, were maintained under standard specific pathogen-free conditions and fed a normal rodent diet (Catalog No. 1025; HFK Bioscience, Beijing, China).

### Experimental design

After two weeks of acclimatization, 24 GK rats (male, 6 weeks old) were randomly divided into four groups: 1) D group (*n* = 6), conventionally raised with distilled water; 2) M group (*n* = 6), administered with metformin (250 mg/kg); 3) GQD group (*n* = 6), administered with GQD (22 g/kg); and 4) BBR group (*n* = 6), administered with BBR (200 mg/kg). For the N group, six nondiabetic Wistar rats (male, 6 weeks old) were intragastrically administered with distilled water. The duration of interventions lasted for 12 weeks. To reduce the cage effect, two rats were kept in each cage. All the drugs were suspended in distilled water for oral gavage once per day. The dosage of GQD was determined based on the content of BBR by high-performance liquid chromatography (HPLC), and we determined that 22 g GQD mixture contained approximately 200 mg of BBR. No treatment (N and D) groups were given the same volume of distilled water to mimic the effects of oral gavage administration. The N, D, and M groups acted as the normal control group, disease model group, and positive control group, respectively.

Body weight and the NFBG level of each rat were monitored twice a week throughout the experimental period. Stool samples were collected at weeks 0 and 12 and then frozen and stored at −80 °C until analysis. After 12 weeks of treatment, all the animals were weighed and anesthetized with diethyl ether. The following specimens were soon taken: 1) serum: abdominal aorta blood was taken and centrifuged for serum collection; 2) pancreatic islets; and 3) rat ileum.

### Oral glucose tolerance test

OGTT was performed 1 week before euthanasia. After 12 h of overnight fasting, all rats were administered oral gavage with glucose solution (2 g/kg). Blood glucose levels were determined at 0 min (fasting glucose, taken before the glucose challenge) as well as 15, 30, 60, and 120 min after glucose administration using an ACCU-CHEK Performa OneTouch glucometer (Catalog No. 06454011; Roche, Mannheim, Germany). AUC for glucose levels was calculated following the trapezoidal rule. Tail vein blood samples at 0 min were simultaneously collected in EDTA-coated tubes to measure insulin concentration (fasting insulin) using ultrasensitive rat insulin ELISA kit (Catalog No. 80-INSRTU-E01; ALPCO, Windham, NH). HOMA-IR was calculated as described previously [56]: HOMA-IR = [insulin (μM) × glucose (mM) /22.5].

### 16S rRNA gene sequencing

Rat stool DNA was extracted from the frozen samples using PowerSoil DNA extraction kit (Catlog No. 12888-100; QIAGEN, Germantown, MD) according to the manufacturer’s instructions. The V3–V4 region of the 16S rRNA gene was amplified by PCR with the primer pair 341F/805R (341F: CCTACGGGNGGCWGCAG; 805R: GACTACHVGGGTATCTAATCC). Sequencing was performed using the Illumina HiSeq 2500 sequencer, which produced partially overlapping 250-bp pair-end reads. Sequence analysis was performed following our previous study [57]. Briefly, the demultiplexed FASTQ sequences were merged using the FLASH program with default parameters [58], and the successfully combined sequences were subjected to quality control using the FASTX-Toolkit (http://hannonlab.cshl.edu/fastx_toolkit/). Chimeras were removed using the USEARCH program’s UCHIME command and the ‘GOLD’ database. After the random rarefication of each sample to 3048 reads, the taxonomical classification of reads was determined using the RDP classifier to generate the composition matrices at the level of the phylum to the genus [59]. A bootstrap value >0.8 was considered the high-confidence taxonomy assignment, while the low-confidence sequences were labeled the unclassified assignment. Genus level tables were created using our in-house Perl scripts.

### Measurements of SCFAs

Levels of fecal SCFAs were measured at LipidALL Technologies Company (Beijing, China) using HPLC coupled with mass spectrometry. In brief, SCFAs were extracted from feces using acetonitrile/water solvent mixtures. Octanoic acid-1-^13^C_1_ was used as an internal standard for accurate quantification of individual SCFAs. Detailed information has been described elsewhere [60].

### RNA-seq analysis of ileum

Total RNA from ileum tissue samples was extracted with TRIzol (Catalog No. DP424; Tiangen Biotech, Beijing, China) according to the standard isolation protocol. RNA integrity and purity were assessed with Agilent Bioanalyzer 2100 (Agilent Technologies, Santa Clara, CA) and quantified using Qubit Fluorometer (Invitrogen, Carlsbad, CA). For analysis, RNA samples had to meet the following quality criteria: RNA integrity number > 7.0 and 28S/18S ratio > 1.8.

The RNA libraries for sequencing were constructed using the NEBNext Ultra RNA Library Prep Kit for Illumina [Catalog No. E7530L; New England Biolabs (NEB), Ipswich, MA] with 1 μg total RNA according to the manufacturer’s protocol. In brief, poly(A) + mRNA was enriched from the total RNA using the NEBNext Poly(A) mRNA Magnetic Isolation Module (Catalog No. E7490L; NEB), and then the purified mRNA was randomly fragmented into sequences approximately 200 bp in length. A cDNA library was obtained with random hexamer primers (Catalog No. SO142; Thermo Fisher Scientific, Waltham, MA) and reverse transcriptase (Catalog No. D2640B; Takara, Dalian, China). After end repair of the cDNA fragments, adaptors were added to the other end of the cDNA products, and then the cDNA library was amplified by PCR. The KAPA Library Quantification Kit (Catalog No. KK4824; KAPA Biosystems, Wilmington, MA) was used to quantify the final libraries with Agilent Bioanalyzer 2100 (Agilent Technologies). After validation by qPCR, libraries were finally sequenced on the Illumina HiSeqXTen platform using the PE150 module.

Quality-controlled clean sequences were mapped to the rat Rnor 6.0 genome using the efficient splice aligner HISAT2 [61]. Aligned paired-end reads were counted at the gene level using the HTSeq program [62]. DEGs were identified using the DESeq2 program [63] with the cutoff threshold of *P* < 0.05 and the absolute value of log_2_ fold change (log_2_ FC) > 1.

GO (http://www.geneontology.org/) and KEGG enrichment analyses (http://www.genome.jp/kegg) were performed using DEGs as the foreground genes and all genes as the background. Significantly enriched GO terms and KEGG pathways were identified with the DEGs identified above using the *clusterProfiler* package [64] with the Benjamini–Hochberg (BH) multiple testing–corrected *P* < 0.05.

### Measurements of serum cytokines

Concentrations of six cytokines (IL-1β, IL-6, IL-17, TNF-α, IFN-γ, and MCP-1) in rat serum were determined using the Luminex Multiplex assay (Catalog No. 12005641; Bio-Rad, Hercules, CA) according to the manufacturer’s protocol. All multiplexing assays were performed on the Bio-Plex MAGPIX Multiplex reader system (Catalog No. 171015001; Bio-Rad). Briefly, serum samples were incubated with capture antibody-coupled magnetic beads. After three washes in a Tecan washing station, the samples were incubated with the biotinylated detection antibody. Each captured cytokine was detected by the addition of streptavidin–phycoerythrin. The standard curve was utilized to convert optical density values into cytokine concentrations (pg/ml).

### Preparation of pancreatic islets and qPCR analysis

Pancreatic islets from GK and Wistar rats were isolated by collagenase digestion and subsequently handpicked in Hank’s buffer under a dissection microscope after separation on a Ficoll density gradient (Catalog No. 17-1140-02; GE Healthcare, Little Chalfont, UK). For qPCR, total RNA was isolated from pancreatic islets using TRIzol reagent (Catalog No. 15596018; Life Technologies, Carlsbad, CA) and then transcribed to cDNA using a reverse transcription kit (Catalog No. DRR047A; Takara). qPCR was performed with technical triplicates using SYBR Green reagent (Catalog No. 10041595; Bio-Rad). The expression levels were calculated with the 2^–△△Ct^ method, and the Ct values were normalized using *Gapdh* as a reference gene. The target genes are *Nfkb1*, *Stat1*, and *Ifngr1*. All primers are listed in Table S1.

### Statistical analysis

All statistical analyses were performed using R software. Data are presented as mean ± SEM. The Kruskal–Wallis or Wilcoxon rank sum test was used for comparisons between groups when appropriate. Bray–Curtis distance was calculated as the beta diversity measurement using the *vegan* package. The PERMANOVA test with the *adonis* package was used to calculate the community structure differences. The *Capscale* package was used to perform the PCoA of all samples based on the Bray–Curtis distance. All figures were created using the *ggplot2*, *gplots*, *TCseq*, or *VennDiagram* package. *P* < 0.05 or BH-adjusted *P* < 0.05 was considered statistically significant.

## Ethical statement

All animal experimental protocols were approved by the Research Ethics Committee of Guang'anmen Hospital, China Academy of Chinese Medical Sciences (IACUC-GAMH-2018-004) and followed the guidelines of laboratory animal care in the Declaration of Helsinki.

## Data availability

All sequencing data have been deposited in the Genome Sequence Archive [65] at the National Genomics Data Center, Beijing Institute of Genomics, Chinese Academy of Sciences / China National Center for Bioinformation (GSA: CRA001199 for 16S rRNA gene sequencing and GSA: CRA001200 for RNA-seq), which are publicly accessible at bigd.big.ac.cn/gsa.

## CRediT author statement

**Xizhan Xu:** Methodology, Software, Data curation, Formal analysis, Visualization, Writing - original draft, Writing - review & editing. **Zezheng Gao:** Methodology, Investigation, Data curation, Resources, Writing - original draft, Writing - review & editing. **Fuquan Yang:** Resources, Supervision. **Yingying Yang:** Investigation. **Liang Chen:** Methodology, Visualization. **Lin Han:** Investigation. **Na Zhao:** Investigation, Project administration. **Jiayue Xu:** Methodology, Visualization. **Xinmiao Wang:** Investigation. **Yue Ma:** Data curation. **Lian Shu:** Investigation. **Xiaoxi Hu:** Formal analysis. **Na Lyu:** Investigation. **Yuanlong Pan:** Investigation. **Baoli Zhu:** Project administration. **Linhua Zhao:** Conceptualization, Supervision, Project administration, Funding acquisition. **Xiaolin Tong:** Conceptualization, Supervision, Project administration, Funding acquisition. **Jun Wang:** Conceptualization, Supervision, Project administration, Funding acquisition, Writing - original draft, Writing - review & editing. All authors read and approved the final manuscript.

## Competing interests

The authors have declared no competing interests.

## Supplementary material

Supplementary Figure S1 **Rat gut microbiota profiling using 16S rRNA gene sequencing before and after different treatments**. **A.** Relative abundance of the 15 most abundant genera in the gut microbiota. The vertical dotted lines separate before (denoted as B in samples) from after (denoted as A in samples) treatment in each group. Three samples (M-A6, BBR-B1, and BBR-A2) were removed due to the lack of sufficient high-quality sequencing data after quality control, and the same holds below. **B.** Firmicutes/Bacteroidetes ratio before and after treatment in five groups. The Wilcoxon rank sum test was used to test the difference in the Firmicutes/Bacteroidetes ratio for each group before and after treatment. *, *P* < 0.05; **, *P* < 0.01. *n* = 6 samples per group. N, normal; D, diabetes; M, metformin; GQD, Gegen Qinlian Decoction; BBR, berberine. uc, unclassified higher taxonomic level.

Supplementary Figure S2 **Alpha diversity changes by different treatments**. Three alpha diversity parameters, that is, number of observed genera (**A**), Chao1 index (**B**), and Shannon evenness index (**C**), were calculated using the R package “*vegan*”. The Wilcoxon rank sum test was used to test the difference in alpha diversity for each group before and after treatment. *, *P* < 0.05; **, *P* < 0.01. *n* = 6 samples per group.

Supplementary Figure S3 **Comparison of gene expression profiles between different treatment groups in the rat ileum**. **A.** PCA showing overall gene expression profiles across all samples. Three rats were randomly selected to collect ileum, and the transcriptomes were profiled by RNA-seq. The first dimension explained 26.94% of the variation along the PC1 and separated N group samples from the other four GK rat samples. The second dimension explained 15.53% of the variation and reflected the biological replicate difference. **B.–K.** Volcano plot showing pairwise comparisons of RNA expression levels highlighting the DEGs. Red dots represent upregulated DEGs (FC > 2), and blue dots represent the downregulated ones (FC < 0.05) with *P* < 0.05. PCA, principal component analysis; FC, fold change.

Supplementary Figure S4 **Functional enrichment of DEGs compared to the normal control group**. **A.** Overrepresented GO terms using upregulated DEGs and downregulated DEGs between indicated groups (D *vs*. N; M *vs*. N; GQD *vs*. N; BBR *vs*. N; BH adjusted *P* < 0.05). **B**. Dotplot shows the enriched KEGG pathways of the corresponding groups. All *P* values were adjusted for multiple testing using the BH method. Adjusted *P* < 0.05 was considered significant. Dot size represents the ratio of the number of DEGs to the number of genes in the corresponding entry. Dots are color-coded according to the adjusted *P* value for each term.

Supplementary Figure S5 **KEGG pathway enrichment analysis of DEGs in terms of cluster or treatment**. **A.** Dotplot for KEGG pathways enriched in the four gene clusters. **B.** Dotplot for KEGG pathways enriched for upregulated DEGs and downregulated DEGs between indicated treatment groups (M *vs*. D; GQD *vs*. D; BBR *vs*. D; GQD *vs*. M; BBR *vs*. M). All *P* values were adjusted for multiple testing using the BH method. Adjusted *P* < 0.05 was considered significant. Dot size represents the ratio of the number of DEGs to the number of genes in the corresponding entry. Dots are color-coded according to the adjusted *P* value for each term.

Supplementary Figure S6 **Functional enrichment of common DEGs shared by GQD vs. D and BBR vs. D comparisons**. **A.** Barplot for the enriched GO terms of all 169 common DEGs shared by GQD *vs*. D and BBR *vs*. D comparisons. **B.** Barplot for the enriched KEGG pathways of all 169 common DEGs shared by GQD *vs*. D and BBR vs. D comparisons. The bar length represents the number of DEGs in the corresponding entry. Bars are color-coded according to the adjusted *P* value for each term.
