# Supplementary figures and images for: Antidiabetic Effects of Gegen Qinlian Decoction via the Gut Microbiota Are Attributable to Its Key Ingredient Berberine
Source: Genomics Proteomics Bioinformatics. 2020 Dec 24;18(6):721–36. doi: 10.1016/j.gpb.2019.09.007 (PMC8377040; doi:10.1016/j.gpb.2019.09.007)

**A**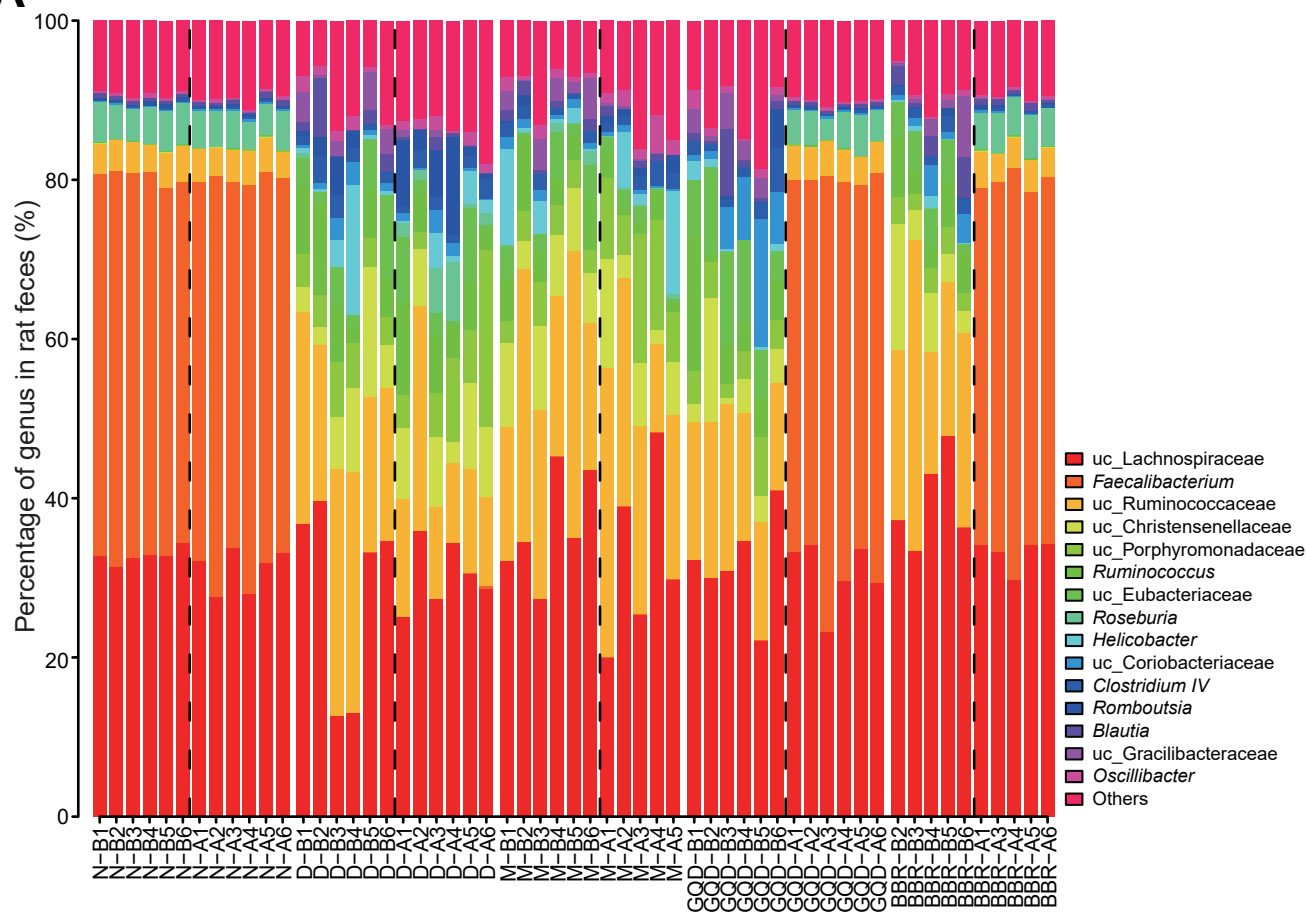**B**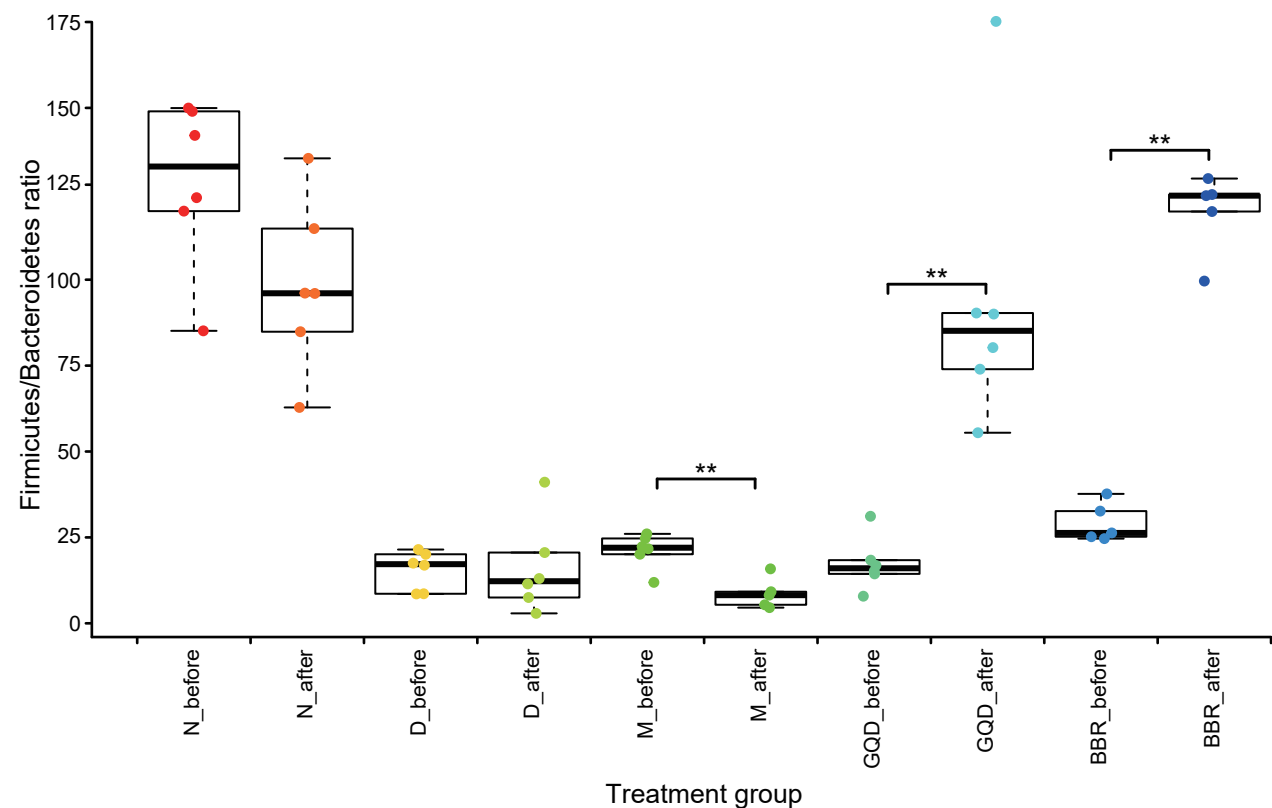

Supplement: Supplementary Figure S1 — Rat gut microbiota profiling using 16S rRNA gene sequencing before and after different treatments. A. Relative abundance of the 15 most abundant genera in the gut microbiota. The vertical dotted lines separate before (denoted as B in samples) from after (denoted as A in samples) treatment in each group. Three samples (M-A6, BBR-B1, and BBR-A2) were removed due to the lack of sufficient high-quality sequencing data after quality control, and the same holds below. B. Firmicutes/Bacteroidetes ratio before and after treatment in five groups. The Wilcoxon rank sum test was used to test the difference in the Firmicutes/Bacteroidetes ratio for each group before and after treatment. *, P < 0.05; **, P < 0.01. n = 6 samples per group. N, normal; D, diabetes; M, metformin; GQD, Gegen Qinlian Decoction; BBR, berberine. uc, unclassified higher taxonomic level. [file mmc1.pdf]

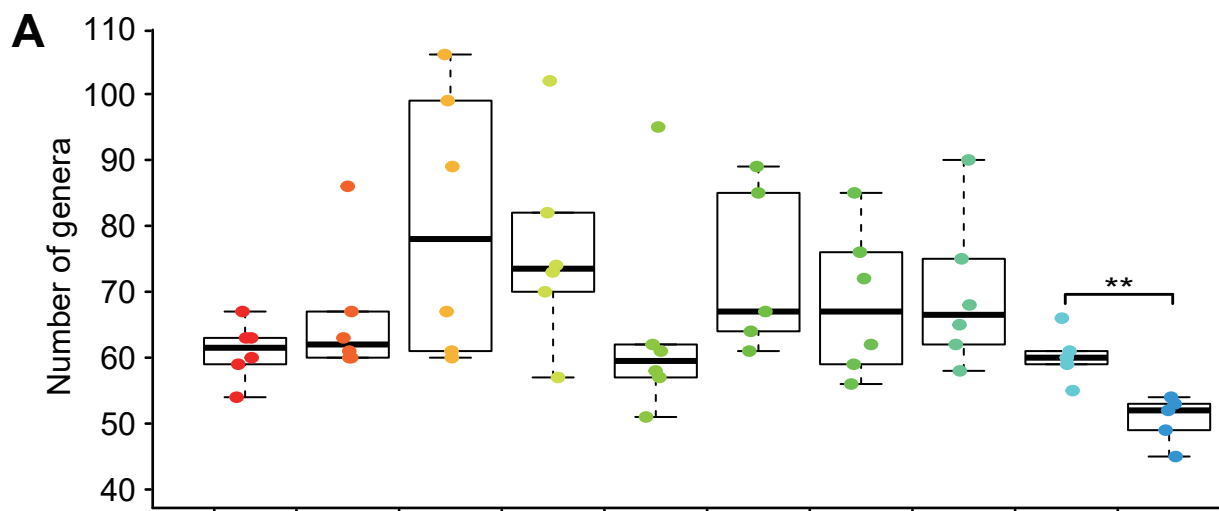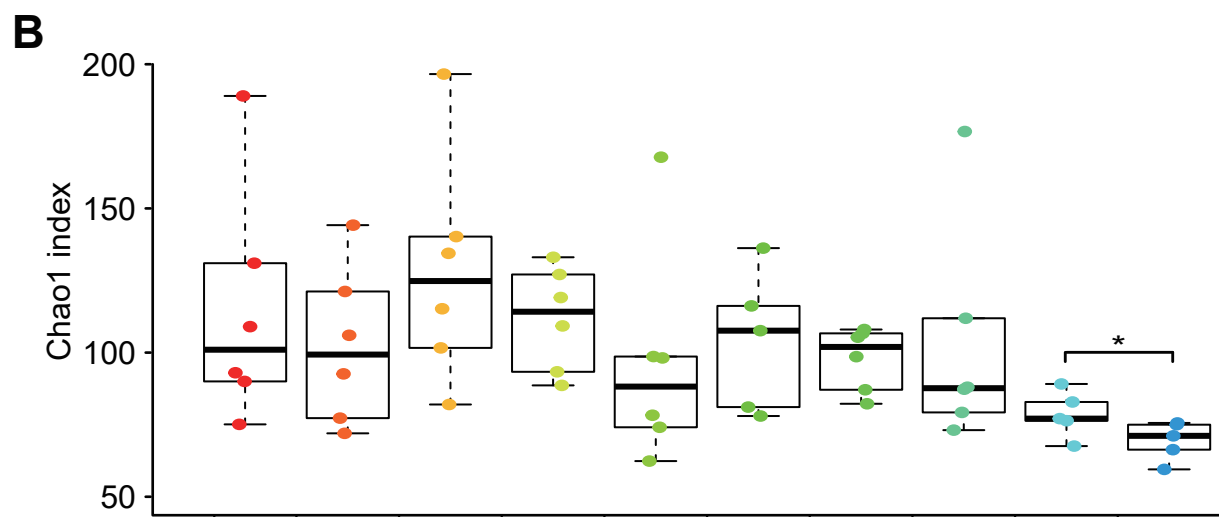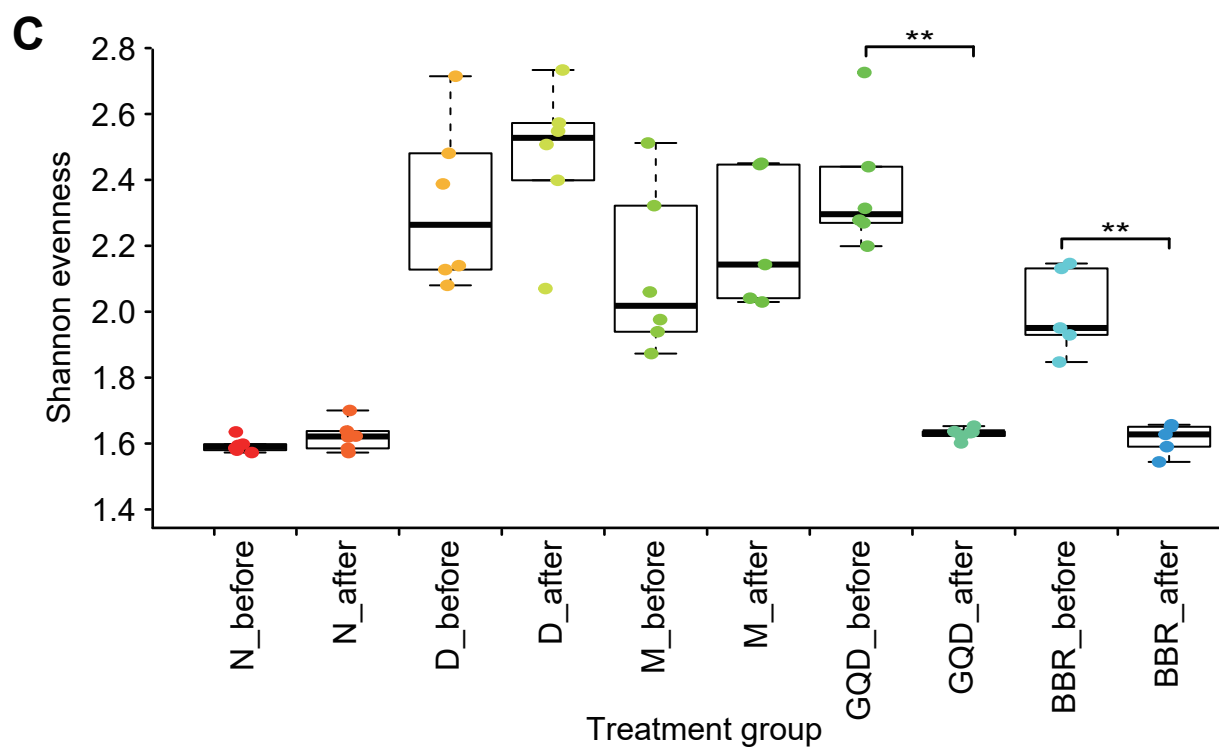

Supplement: Supplementary Figure S2 — Alpha diversity changes by different treatments. Three alpha diversity parameters, that is, number of observed genera (A), Chao1 index (B), and Shannon evenness index (C), were calculated using the R package “vegan”. The Wilcoxon rank sum test was used to test the difference in alpha diversity for each group before and after treatment. *, P < 0.05; **, P < 0.01. n = 6 samples per group. [file mmc2.pdf]

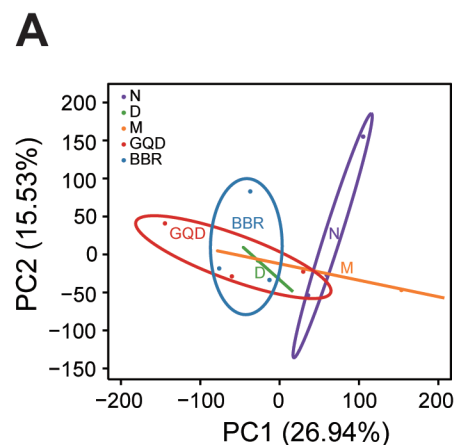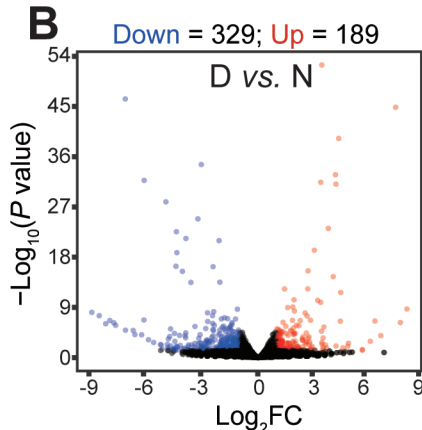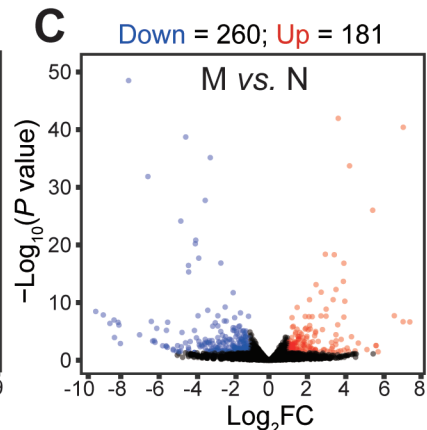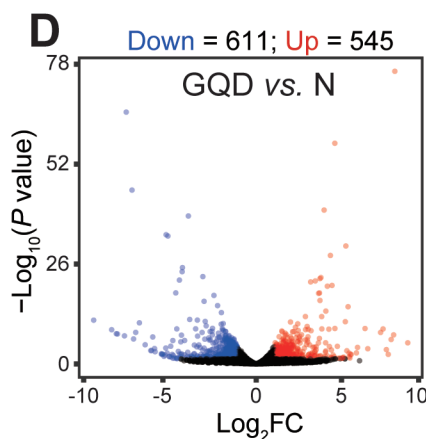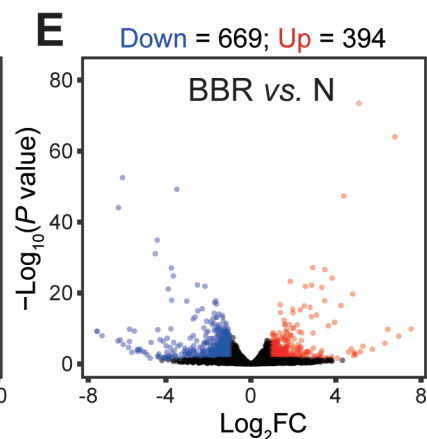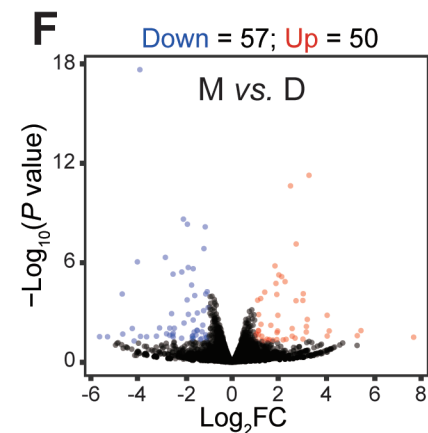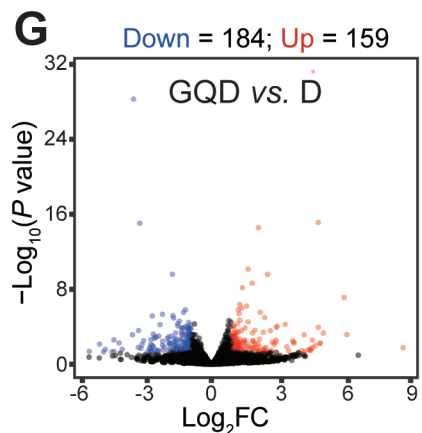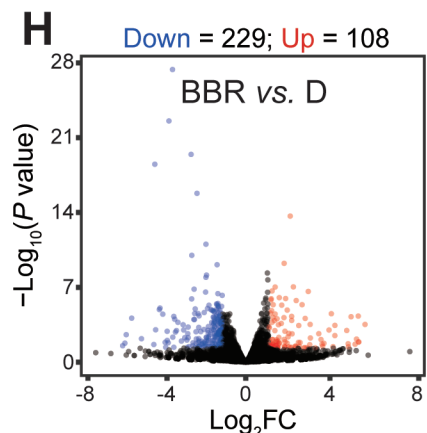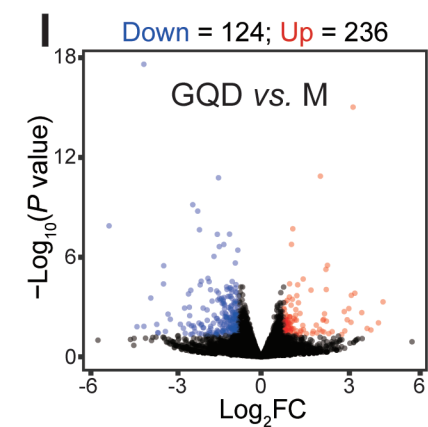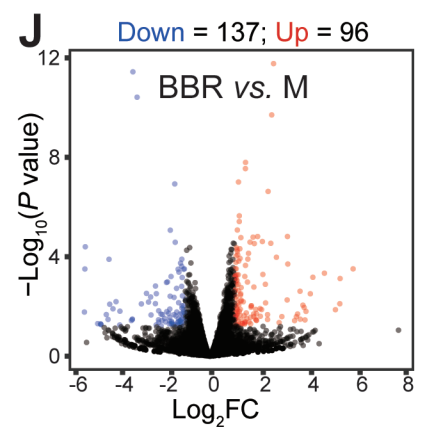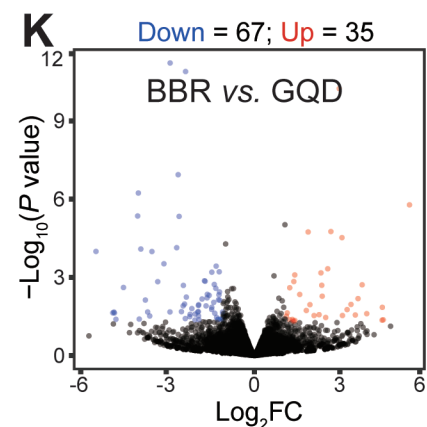

Supplement: Supplementary Figure S3 — Comparison of gene expression profiles between different treatment groups in the rat ileum. A. PCA showing overall gene expression profiles across all samples. Three rats were randomly selected to collect ileum, and the transcriptomes were profiled by RNA-seq. The first dimension explained 26.94% of the variation along the PC1 and separated N group samples from the other four GK rat samples. The second dimension explained 15.53% of the variation and reflected the biological replicate difference. B.–K. Volcano plot showing pairwise comparisons of RNA expression levels highlighting the DEGs. Red dots represent upregulated DEGs (FC > 2), and blue dots represent the downregulated ones (FC < 0.05) with P < 0.05. PCA, principal component analysis; FC, fold change. [file mmc3.pdf]

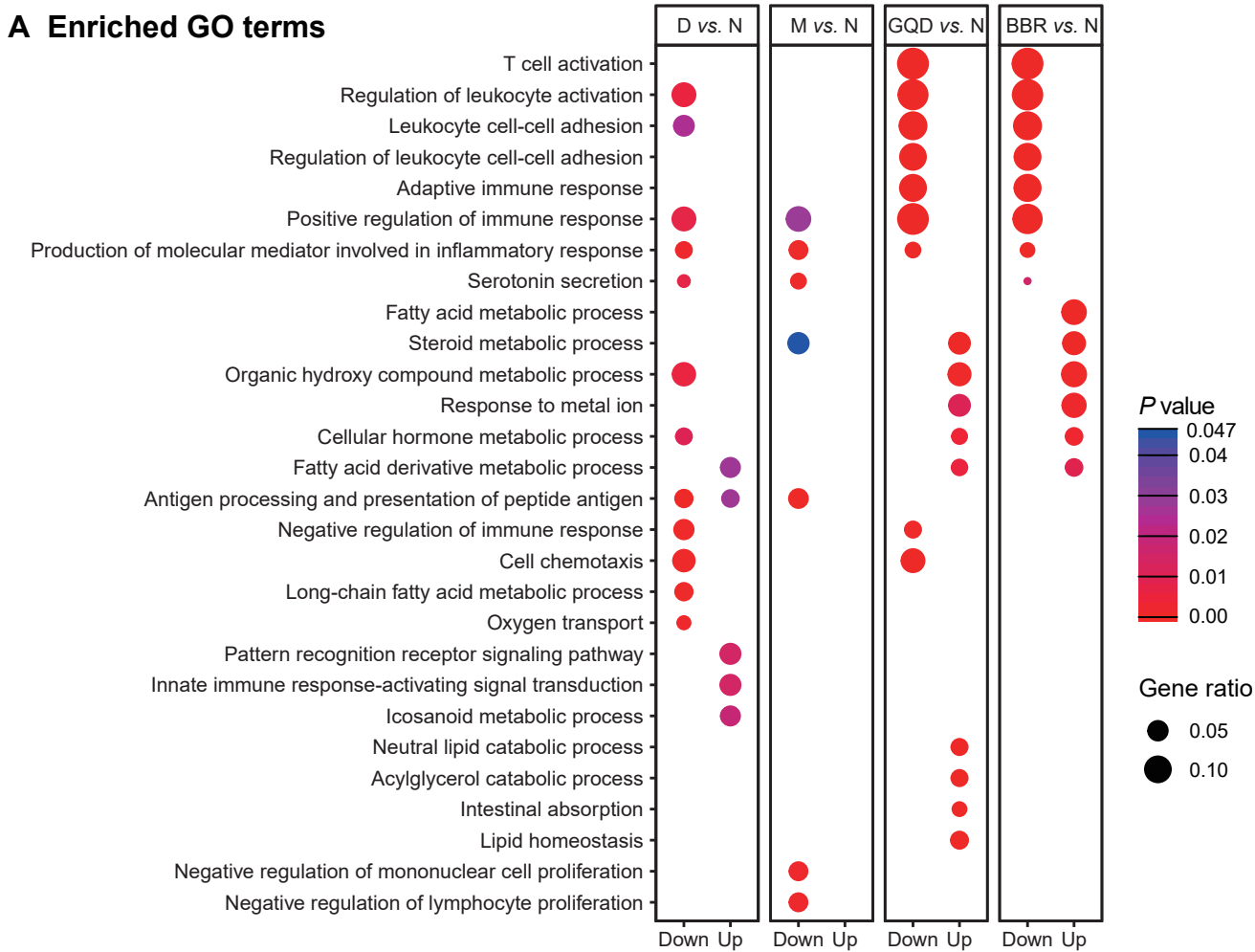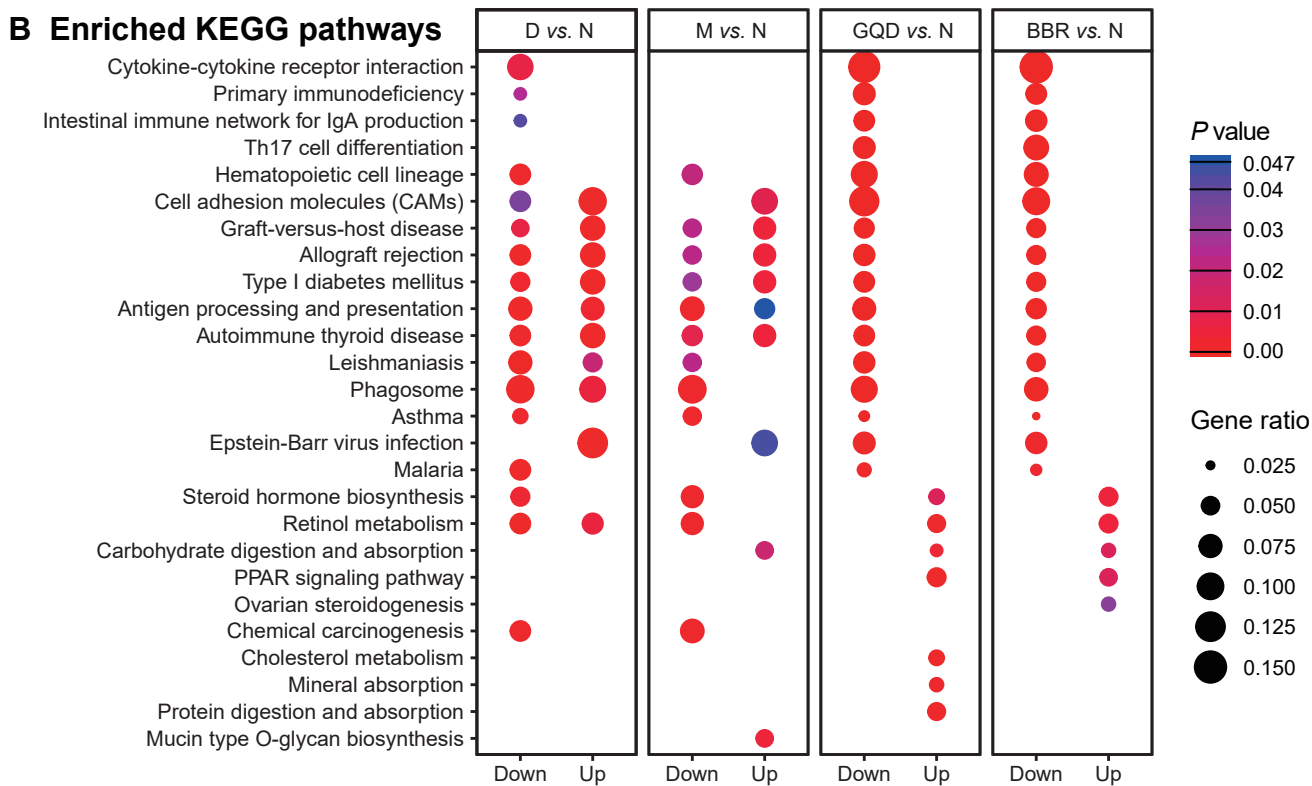

Supplement: Supplementary Figure S4 — Functional enrichment of DEGs compared to the normal control group. A. Overrepresented GO terms using upregulated DEGs and downregulated DEGs between indicated groups (D vs. N; M vs. N; GQD vs. N; BBR vs. N; BH adjusted P < 0.05). B. Dotplot shows the enriched KEGG pathways of the corresponding groups. All P values were adjusted for multiple testing using the BH method. Adjusted P < 0.05 was considered significant. Dot size represents the ratio of the number of DEGs to the number of genes in the corresponding entry. Dots are color-coded according to the adjusted P value for each term. [file mmc4.pdf]

## A Enriched KEGG cell signaling pathways

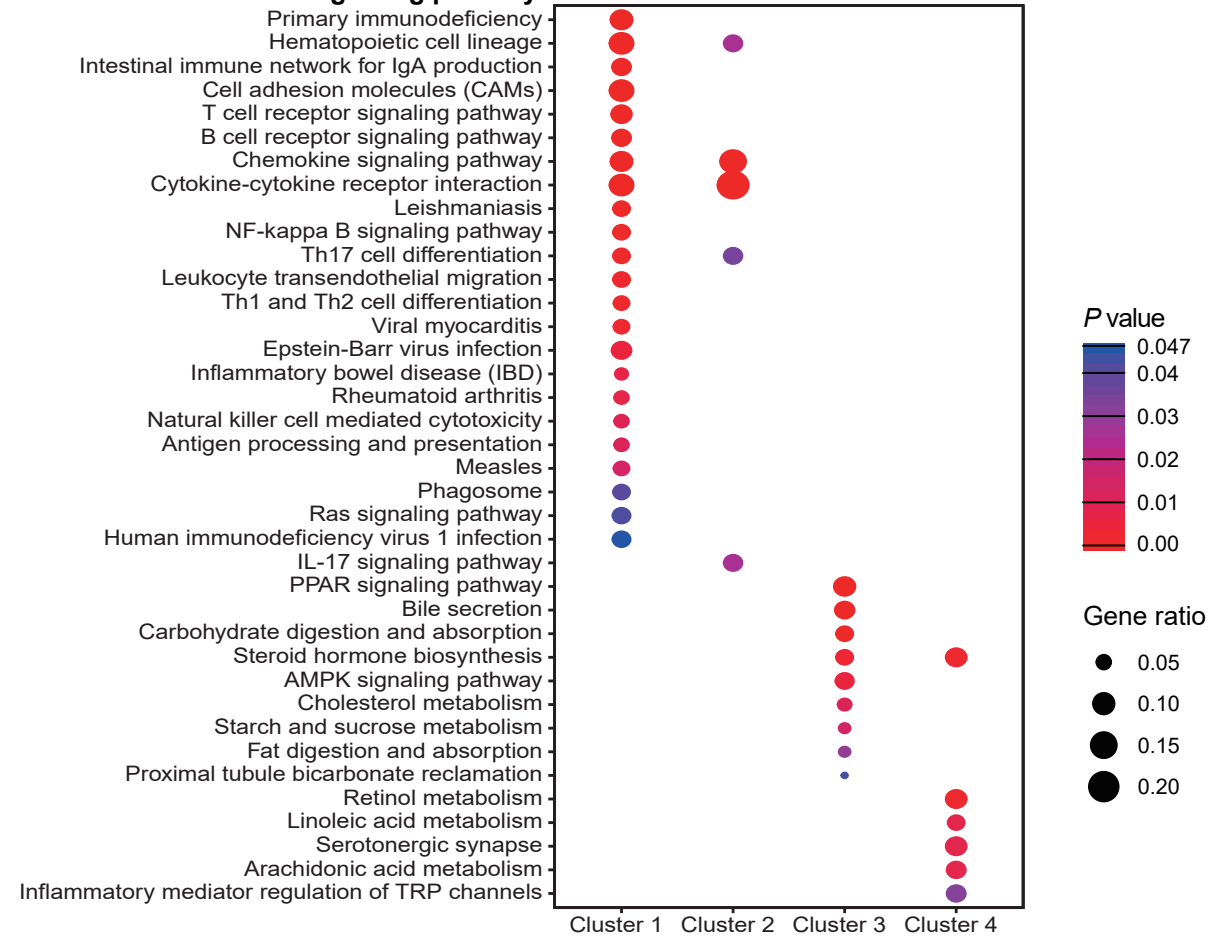

## B Enriched KEGG metabolic pathways

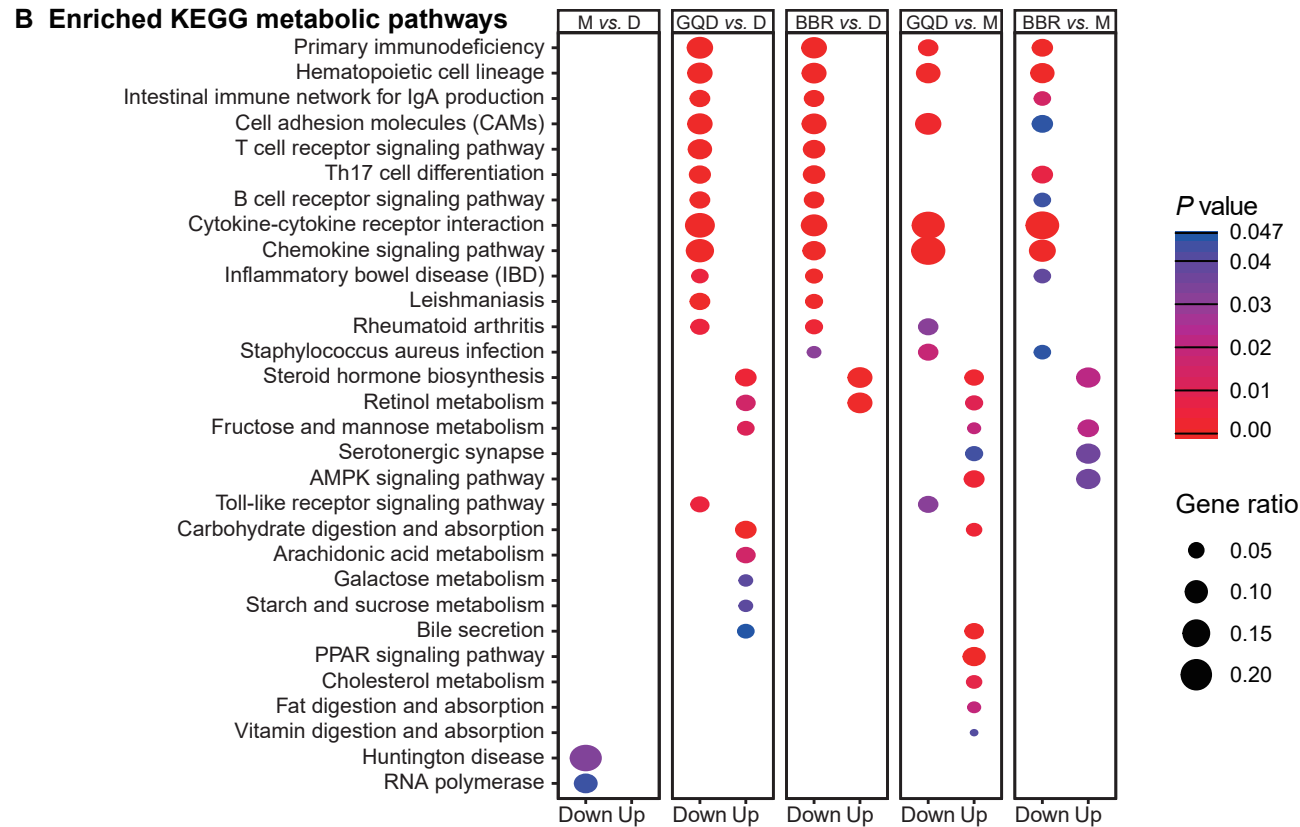

Supplement: Supplementary Figure S5 — KEGG pathway enrichment analysis of DEGs in terms of cluster or treatment. A. Dotplot for KEGG pathways enriched in the four gene clusters. B. Dotplot for KEGG pathways enriched for upregulated DEGs and downregulated DEGs between indicated treatment groups (M vs. D; GQD vs. D; BBR vs. D; GQD vs. M; BBR vs. M). All P values were adjusted for multiple testing using the BH method. Adjusted P < 0.05 was considered significant. Dot size represents the ratio of the number of DEGs to the number of genes in the corresponding entry. Dots are color-coded according to the adjusted P value for each term. [file mmc5.pdf]

## A Enriched GO terms

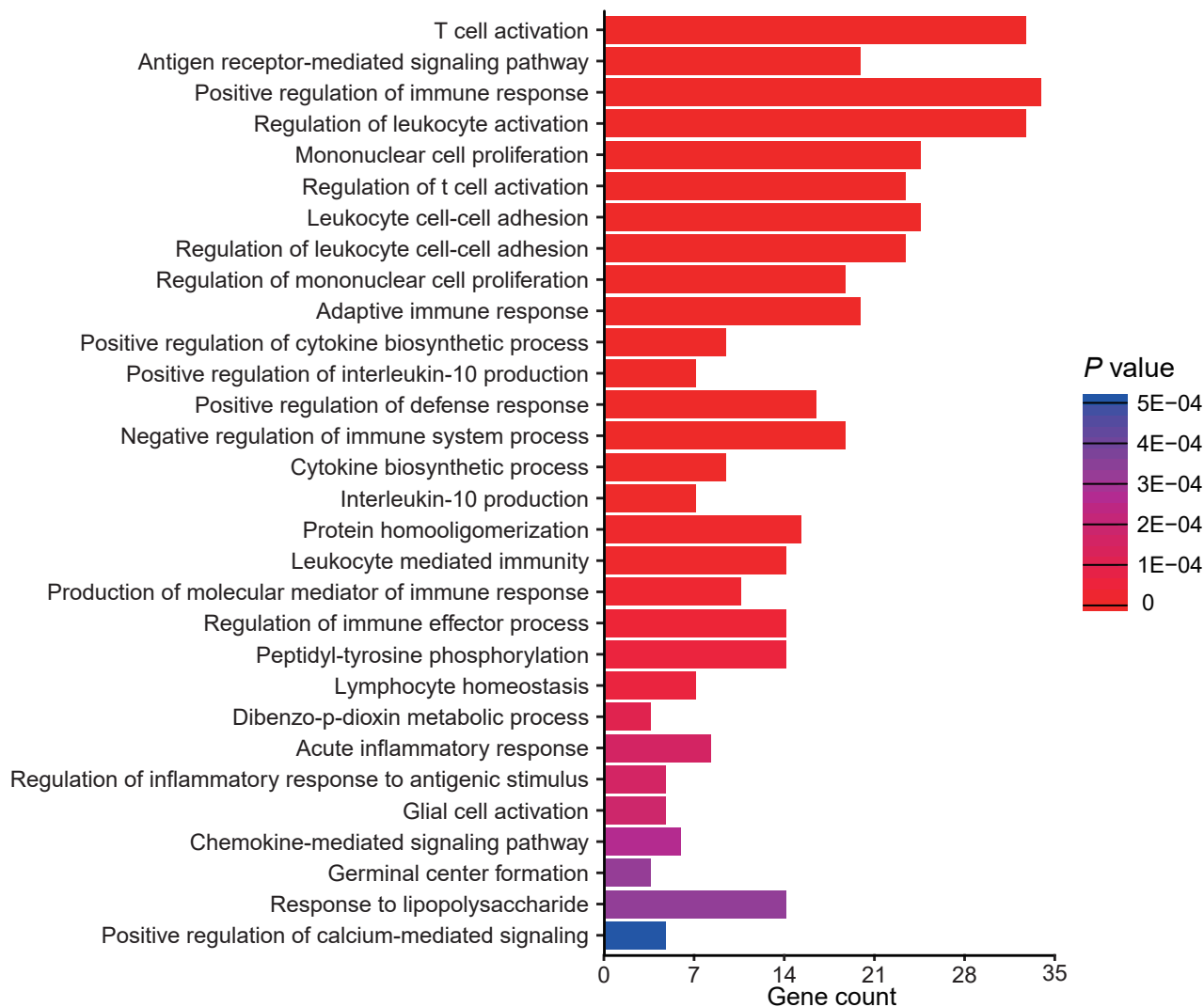

## B Enriched KEGG pathways

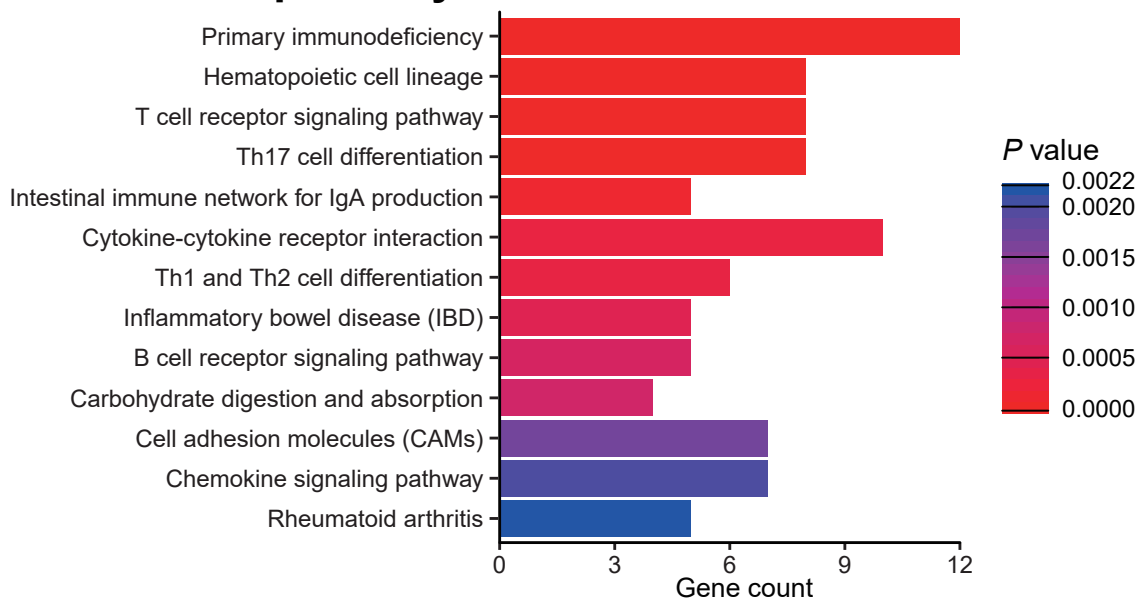

Supplement: Supplementary Figure S6 — Functional enrichment of common DEGs shared by GQD vs. D and BBR vs. D comparisons. A. Barplot for the enriched GO terms of all 169 common DEGs shared by GQD vs. D and BBR vs. D comparisons. B. Barplot for the enriched KEGG pathways of all 169 common DEGs shared by GQD vs. D and BBR vs. D comparisons. The bar length represents the number of DEGs in the corresponding entry. Bars are color-coded according to the adjusted P value for each term. [file mmc6.pdf]
